# Comparison and evaluation of pathway-level aggregation methods of gene expression data

**DOI:** 10.1186/1471-2164-13-S7-S26

**Published:** 2012-12-07

**Authors:** Seungwoo Hwang

**Affiliations:** 1 Korean Bioinformation Center, Korea Research Institute of Bioscience and Biotechnology, Daejeon, Korea

## Abstract

**Background:**

Microarray experiments produce expression measurements in genomic scale. A way to derive functional understanding of the data is to focus on functional sets of genes, such as pathways, instead of individual genes. While a common practice for the pathway-level analysis has been functional enrichment analysis such as over-representation analysis and gene set enrichment analysis, an alternative approach has also been explored. In this approach, gene expression data are first aggregated at pathway level to transform the original data into a compact representation in which each row corresponds to a pathway instead of a gene. Thereafter the pathway expression data can be used for differential expression and classification analyses in pathway space, leveraging existing algorithms usually applied to gene expression data. While several studies have proposed the pathway-level aggregation methods, it remains unclear how they compare with one another, since the evaluations were done to a limited extent. Thus this study presents a comprehensive evaluation of six most prominent aggregation methods.

**Results:**

The compared methods include five existing methods--mean of all member genes (*Mean all*), mean of condition-responsive genes (*Mean CORGs*), analysis of sample set enrichment scores (ASSESS), principal component analysis (PCA), and partial least squares (PLS)--and a variant of an existing method (*Mean top 50%*, averaging top half of member genes). Comprehensive and stringent benchmarking was performed by collecting seven pairs of related but independent datasets encompassing various phenotypes. Aggregation was done in the space of KEGG pathways. Performance of the methods was assessed by classification accuracy validated both internally and externally, and by examining the correlative extent of pathway signatures between the dataset pairs. The assessment revealed that (i) the best accuracy and correlation were obtained from ASSESS and *Mean top 50%*, (ii) *Mean all *showed the lowest accuracy, and (iii) *Mean CORGs *and PLS gave rise to the largest extent of discordance in the pathway signature correlation.

**Conclusions:**

The two best performing method (ASSESS and *Mean top 50%*) are suggested to be preferred. The benchmarking analysis also suggests that there is both room and necessity for developing a novel method for pathway-level aggregation.

## Background

Microarray gene expression experiments produce high dimensional expression measurements in genomic scale, typically expression levels of more than ten thousand genes. While the high dimensionality offers an opportunity for a comprehensive interrogation of transcriptome, it also poses a challenge to researchers seeking to extract functional understanding and interpretation of the data, which go beyond a mere identification of differentially expressed genes (DEGs). A way to derive such a functional interpretation is to focus on functional sets of genes, such as biological pathways, instead of individual genes. A common practice for the pathway-level analysis has been the functional enrichment analysis, for which two types of methods are widely used. One is called over-representation analysis in which DEGs are first identified from the data and their prevalent pathway annotations are then identified, whose best known example is DAVID [1]. The other is called gene set analysis in which differential expression statistics of all genes are first computed and each pathway is then examined to see whether its member genes collectively show significant differential expression, whose best known example is Gene Set Enrichment Analysis (GSEA) [2]. A common theme underlying the two types of functional enrichment analysis is that individual genes are first tested for differential expression and the resultant gene-level results are then combined at the pathway level for an identification of differentially regulated pathways.

An alternative approach is also possible, where gene expression data are first aggregated at pathway level to yield a compact representation of the original data, in which each row in the data matrix now corresponds to a pathway instead of a gene. Thereafter the pathway-level aggregated expression data, or simply, pathway expression data, are directly analyzed to identify differentially expressed pathways. A notable advantage of the aggregation-based approach is that it can be applied to a wider range of analysis tasks than the conventional functional enrichment analysis. This is because, once gene expression data are aggregated at pathway level, the pathway expression data can not only be used for identifying differentially expressed pathways, but also for classification or clustering of samples in the space of pathways [3,4], leveraging existing algorithms usually applied to gene expression data. Transforming the expression data from gene space to pathway space is also expected to yield a more robust representation of the data in which intrinsic technological and biological variances across samples are reduced [5]. In other words, while expression of individual genes in a pathway may vary considerably across samples with similar phenotypic characteristics, expression of the pathway as a whole may become consistent across the samples. Noting the advantages in interpretability, compactness, utility, and anticipated robustness, several studies have proposed pathway-level aggregation methods of gene expression data [3,5-10].

With the availability of all these pathway-level aggregation methods, it becomes important to comprehensively evaluate how these methods compare with one another. Although each of the original reports has presented an evaluation of its performance for a demonstration of its improvements over existing methods, the evaluations were done only to a limited extent and thus inadequate to make general recommendations. Most notably, there were the following two major limitations.

The first limitation is that, in the studies in which classification accuracy was assessed in the pathway space [3,5,8,9], external validation of accuracy on related but independent test datasets was either skipped (employing internal cross-validation only) [5], or insufficiently done on a limited number of datasets (only two pairs of training and test datasets) [3,8,9]. Although internal cross-validation is a convenient solution to assess a classifier's performance within a single dataset, it leads to optimistically higher estimates of performance than what would really be expected in a new dataset with identical phenotypic classes [11,12]. Thus, in any classification studies, it is increasingly being recognized that it is crucial to externally validate the performance of a classifier constructed from a dataset on an independent test dataset for a realistic estimation of classification performance and generalizability [11,12]. In addition, to ensure a comprehensive evaluation, the training and the external validation need to be performed on multiple pairs of training and test datasets that encompass a wide range of phenotypes with varying extent of relative subtlety in the biological differences between phenotypic classes.

The second limitation is that all the original reports did not evaluate the extent to which the differential expression signature of pathways is correlated between datasets with identical phenotypic classes. As described earlier, an expectation with the pathway-level aggregation has been that, although differential expression signature of genes may show some discrepancy between related datasets, differential expression signature of pathways may become more congruent with each other. Despite the importance as an evaluation metric, the correlative extent of pathway signature has not been evaluated in the original reports.

To address the aforementioned limitations towards a reliable assessment, this study presents a comprehensive evaluation of six most prominent pathway-level aggregation methods of gene expression data--five existing methods and a simple variant of an existing method. Datasets collected for benchmarking consist of seven pairs of two-class gene expression datasets (fourteen datasets in total) of various phenotypes. The gene expression datasets were aggregated in the space of KEGG pathways. The performance of the pathway-level aggregation methods was assessed by classification accuracy validated both internally and externally, and by correlation of pathway signatures.

## Results

### Description of six compared methods of pathway-level aggregation

**Figure 1:**
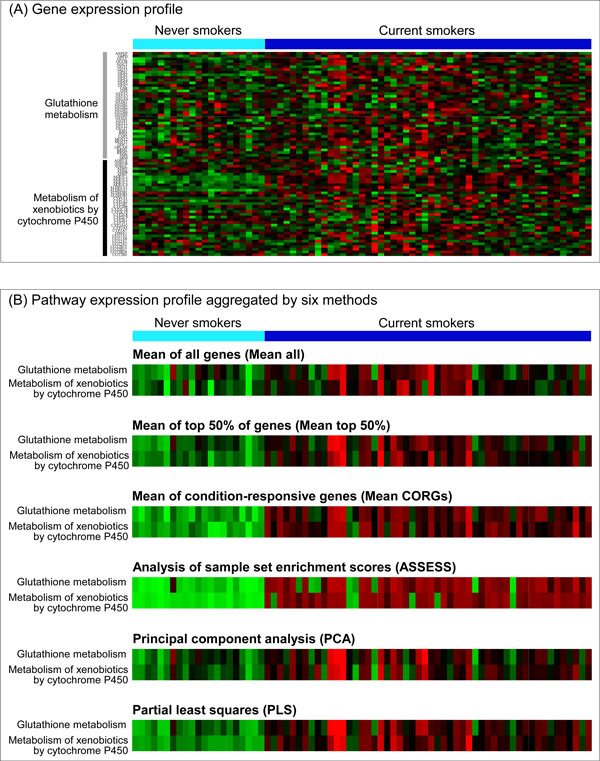
**Illustration of pathway-level aggregation of gene expression data**. (A) A gene expression data in which the effect of smoking on bronchial epithelium was investigated by comparing current smokers and never smokers (the Beane dataset in Table 1). To save space, expression levels of 76 selected genes are shown, which are the member genes of two major pathways mediating the effect of smoking. (B) Corresponding pathway expression profiles aggregated by the six compared methods. With this compact representation of the original gene-level data, it becomes easier to recognize that the two pathways are up-regulated by smoking.

This study compared six of the most prominent methods for pathway-level aggregation of gene expression data. For an illustrative purpose, an example of a gene expression profile and corresponding pathway expression profiles aggregated by the six methods is provided as heatmaps in Figure 1. The six methods can be grouped into three general categories: mean-based (mean of all genes, mean of condition-responsive genes, and mean of top 50% of genes), projection-based (principal component analysis and partial least squares), and others (analysis of sample set enrichment scores). Description of each of the methods is given below. Mathematical description of the methods is provided in Additional file 1.

#### Mean of all genes (Mean all)

In this most straightforward method, which has appeared in the literature many times in one form or the other [4,9,10,13,14], the gene expression data are first *z*-scaled for each gene across the samples into zero mean and unit variance. Then the expression profiles of all member genes in a pathway are combined by taking their mean.

#### Mean of condition-responsive genes (Mean CORGs)

In this method [8], expression profile of a pathway is represented by the mean expression of key member genes, called CORGs (condition-responsive genes), instead of all the member genes. The CORGs are defined as the genes that, upon aggregating their expression profiles by averaging, yield a pathway expression profile that is the most discriminative between the two classes in the data. Identifying the CORGs of a pathway in a given two-class dataset begins with *t*-test on *z*-scaled expression data of genes belonging to the given pathway. Then, overall direction of expression change (that is, up or down) in the pathway is determined as the sign of the mean *t*-statistic values of all its member genes. Next, all the member genes are sorted by their *t*-statistic values in accordance with the overall regulatory direction of the pathway; the most strongly up-regulated genes are arranged to the top for an overall up-regulated pathway, whereas the most strongly down-regulated genes are arranged to the top for an overall down-regulated pathway. Then, the CORG set is initially set to contain only the top ranked gene, and iteratively expanded. At each iteration, the gene of the next rank is added to the candidate CORG set, pathway expression profile is obtained next by taking the mean of expression profiles of the candidate CORGs, and *t*-test is then performed to give the pathway's *t*-statistic. The iteration stops when the pathway's *t*-statistic no longer improves, at which point the final CORG set is obtained. Although this method has been called as PAC (Pathway Activity inference using Condition-responsive genes) in the original article, it will be referred to as *Mean CORGs *in this report in order to emphasize the mean-based nature of the method as well as to avoid confusion with PCA (principal component analysis) which is one of the methods evaluated in this article.

#### Analysis of sample set enrichment cores (ASSESS)

The ASSESS method [3] can be considered as a sample-level extension of Gene Set Enrichment Analysis (GSEA) [2]. As in the GSEA method, ASSESS calculates enrichment score of each pathway. The difference is that, while GSEA gives only an overall enrichment score of a pathway between two classes of samples, ASSESS provides an enrichment score of the pathway for each sample. To this end, ASSESS employs random walk computations twice. The first use of random walk is applied at the level of individual genes. Given an expression level of a gene in a sample, the sample's log likelihood ratio of belonging to one class instead of the other is calculated. To calculate the log likelihood ratio, it is needed to first calculate two probabilities that the expression level of a gene in a sample is representative of class 1 or class 2. Random walk is used for this probability calculation. More detailed description of the first use of random walk can be found in the original publication [3]. Then the second use of random walk is done at the level of each pathway. Using the log likelihood ratio values obtained for its member genes, it computes enrichment score of a pathway in a sample by the maximum deviation of the random walk from zero, as in GSEA.

#### Principal component analysis (PCA)

PCA has long been applied to the analysis of gene expression data, especially for an exploratory data visualization to discriminate between sample groups. In this usage of PCA, correlation matrix is first computed from *z*-scaled gene expression data. Each element in the correlation matrix represents a measure of dependencies between corresponding gene pairs, with zero indicates independence. Then, through eigen-decomposition of the correlation matrix, major directions in the data with the largest variability are identified as eigenvectors corresponding to the largest eigenvalues of the correlation matrix. Or, equivalently, the eigenvectors and eigenvalues of the correlation matrix can also be found by singular value decomposition (SVD) of the gene expression matrix itself. The eigenvectors are called the principal components (PCs), or metagenes in gene expression studies. Finally, the gene expression data are projected onto a small number of PCs, usually two or three, and the projections are used for an exploratory visualization of the data. The matrix of PCs is commonly referred to as loadings, in which each column gives the location of each PC axis relative to the original system of axes. The matrix of projections is also commonly referred to as scores, in which each column gives the location of samples with respect to each PC axis.

In addition to its use in the exploratory data visualization, PCA has also been used as a pathway-level aggregation method in several studies [6,7,15]. In this usage, PCs are found by applying PCA to the matrix of *z*-scaled expression levels of member genes in a given pathway, instead of all genes represented in the microarray. Projection of the gene expression data of the pathway onto the first PC is taken as the expression profile of that pathway. In this application of PCA as a pathway-level aggregation method, it is worth mentioning that there exists an issue called sign ambiguity [16], which is an inherent but often overlooked aspect of PCA despite its wide usage in bioinformatics. The sign ambiguity refers to an intrinsic property of PCA and SVD in which the orientations of PCs, or equivalently, those of singular vectors, cannot be mathematically determined. To see why, consider the decomposition of a data matrix **X, X **= **UΣV^T^**, in which **Σ **is a diagonal matrix and the columns of **U **and **V **are the left and right singular vectors, respectively. In this decomposition, the orientation of any right singular vector can be flipped as long as the orientation of the corresponding left singular vector is flipped as well. In other words, the following equality holds for any column index *k*; $\sigma_{k}u_{k}v_{k}^{\text{T}}=\sigma_{k}\left(-u_{k}\right){\left(-v_{k}\right)}^{\text{T}}$. Although any implementation of PCA and SVD assigns singular vectors with specific signs, the assignment of sign is essentially random [16]. What this entails in the context of pathway-level aggregation is that, for a pathway expression profile obtained from PCA, positive and negative expression values may not necessarily mean an up-regulation and a down-regulation of the pathway, respectively. Thus, the sign of PC scores lacks meaningful biological interpretation. While the sign ambiguity problem has not been recognized in some studies that used PCA as a pathway-level aggregation method [6,7], it was considered in other related studies [15,17] by correcting the signs of PC scores so that the PC scores are positively correlated with the average gene expression profile of the given module. This simple approach for sign correction was adopted for the analysis shown in this report.

#### Partial least squares (PLS)

PLS is a regression method that combines properties of multiple regression and PCA. Data for PLS analysis consist of a data matrix **X **and a response matrix **Y**, which contain values of the independent and the dependent variables, respectively. In brief, PLS seeks to find latent variables (as opposed to observed variables) that best summarize the variance in the original data **X **and are the most relevant for the response **Y **as well. Unlike the standard multiple regression which builds a regression model between the original data **X **and **Y**, PLS seeks to build a regression model between the latent component scores of **X **and those of **Y**. Unlike PCA which chooses the PCs so that only the variability within **X **is best described, PLS chooses the latent components so that covariance between **X **and the response **Y **is best described. A previous study [6] has used PLS as a pathway-level aggregation method. In this approach, data consist of a matrix **X **of *z*-scaled expression levels of a given pathway's member genes and a class vector **Y**. Each element in the class vector simply indicates class membership of the corresponding sample. Score vector of the first latent component is taken as the expression profile of that pathway. As in the case of PCA, the sign of the latent components needs to receive further consideration. In the previous study [6], a dummy coding scheme was used to represent the class vector, 0 for a control sample and 1 for a case sample. However, the sign of the latent components obtained under this coding scheme lacks meaningful biological interpretation. Since 1 is a larger numeric value than 0, regression on these two dummy numeric values makes the resultant pathway expression levels larger in case samples and smaller in control samples. Thus subsequent differential expression analysis at pathway level would falsely identify all pathways as being up-regulated. Thus, for the analysis shown in this report, the sign of the latent component scores was corrected so that the scores are positively correlated with average gene expression profile of the given pathway--the same correction scheme used for PCA. This correction scheme is also equivalent to the use of 0/1 coding for an overall up-regulated pathway and 1/0 coding for an overall down-regulated pathway.

#### Mean of top 50% of genes (Mean top 50%)

In addition to the five existing methods described above, a simple variant of the first method (mean of all genes) was proposed in this report. In this modification, pathway expression profile is calculated by averaging only the top half of the member genes with larger *t*-statistics, instead of all member genes.

### Collection of benchmarking datasets

For a comprehensive evaluation, a total of seven pairs of independent datasets of various phenotypes was collected, fourteen in total (Table 1). Each dataset will be referred to by the name of the first author in the corresponding article, or by the name of cohort. In all the datasets, class 2 represents a more malignant sample group than class 1. Thus class 2 was designated as the case group and class 1 as the control group.

**Table 1:** Seven pairs (fourteen in total) of independent microarray datasets used for benchmarking

Phenotype	Dataset name and reference	Class 1 (control group) samples	Class 2 (case group) samples	Data source	Platform
Effect of smoking on bronchial epithelium		Never smokers	Current smokers		
				
	Beane [18]	21	52	GSE7895	U133A
	Vanni [19]	22	37	GSE10135	U133 Plus 2

Subtypes of non-small cell lung cancer (NSCLC)		AD (adenocarcinoma)	SCC (squamous cell carcinoma)		
				
	Bild [20]	58	53	GSE3141	U133 Plus 2
	Lee [21]	63	75	GSE8894	U133 Plus 2

Subtypes of primary high grade glioma		AA (anaplastic astrocytoma)	GBM (glioblastoma multiforme)		
				
	Phillips [22]	21	56	GSE4271	U133 Set
	Sun [23]	19	77	GSE4290	U133 Plus 2

Estrogen receptor (ER) status in breast cancer		ER-negative	ER-positive		
				
	Chin [24]	46	84	E-TABM-158	U133A
	Minn [25]	42	57	GSE2603	U133A

Breast cancer grade		Grade 1	Grade 3		
				
	Desmedt [26]	30	83	GSE7390	U133A
	Sotiriou [27]	28	32	GSE2990	U133A

Lung cancer grade		Grade 1	Grade 3		
				
	Dana-Farber [28]	13	37	Author's website	U133A
	Michigan [28]	26	66		

Clear cell renal cell carcinoma (CCRCC) vs Normal kidney		Normal kidney	Tumorous kidney		
				
	Jones [29]	23	32	GSE15641	U133A
	Kort [30]	12	10	GSE11024	U133 Plus 2

Each of the datasets was referred to in the main text by the dataset name, which is the first author's name or cohort name. Numbers in the table indicate the number of samples.

### Internal cross-validation accuracies in each dataset

**Figure 2:**
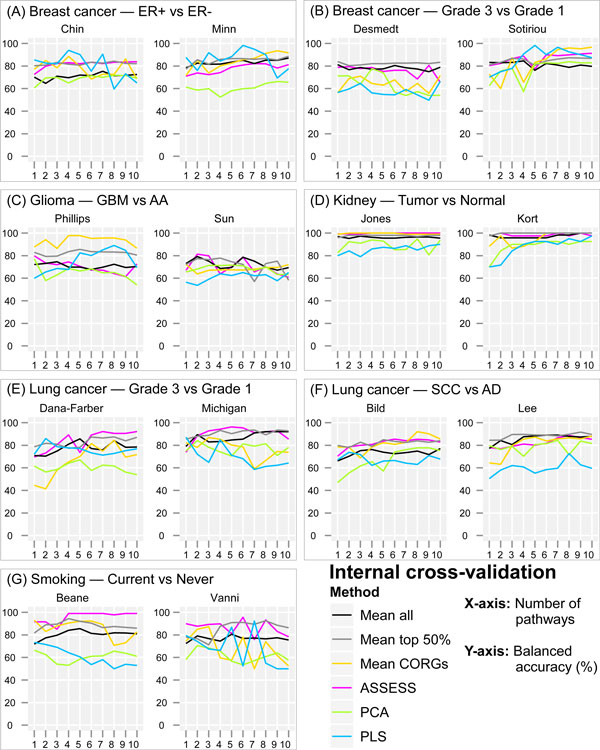
**Internal cross-validation balanced accuracy in the fourteen datasets**. Balanced accuracy is plotted as a function of the number of top pathways added to the classifier, ordered by *t*-test on pathway-level expression data aggregated by the six compared methods.

The ability of pathway expression profile for discriminating two sample groups was first assessed by 5-fold cross-validation within each dataset (Figure 2). Balanced accuracy was examined as a function of feature set size (from top one to ten pathways, ranked by *t*-test on pathway-level aggregated data), instead of at a fixed arbitrary number of features, or an optimized number of features that results in the best performance in the validation dataset [8]. The adopted examination scheme allows a transparent benchmarking of the compared methods over an unbiased operative range, and is an often adopted scheme in similar benchmarking analyses [8,31].

Several observations can be made from Figure 2. First, the classification accuracy varied across datasets, which reflects the relative subtlety in the biological differences between two classes in each dataset. The highest (around 70%-100%) accuracies were obtained in the comparison of tumorous kidney and normal kidney (Figure 2D), which are markedly distinctive disease states. Somewhat lower accuracies were obtained in the remaining datasets. The lowest accuracies (around 55%-80%) were obtained in the Sun dataset (Figure 2C, right) which compared glioblastoma multiforme (GBM) and anaplastic astrocytoma (AA)--the two subtypes of high grade gliomas and also known as grade IV and III astrocytoma, respectively. Second, there were noticeable fluctuations in accuracies along the feature set sizes, and their extent appeared to vary across datasets. For example, the smoking dataset of Vanni showed the largest fluctuations (Figure 2G) whereas the kidney dataset showed the smallest fluctuations (Figure 2D). With respect to aggregation methods, it appears that there are more pronounced fluctuations in some of the methods (*Mean CORGs*, PCA, and PLS) than in the rest (*Mean all, Mean top 50%*, and ASSESS). Third, some of the methods can be recognized to yield lower accuracies than the others. For example, PCA never achieved the highest accuracies across all datasets and feature set sizes.

### External validation accuracies in each pair of datasets

**Figure 3:**
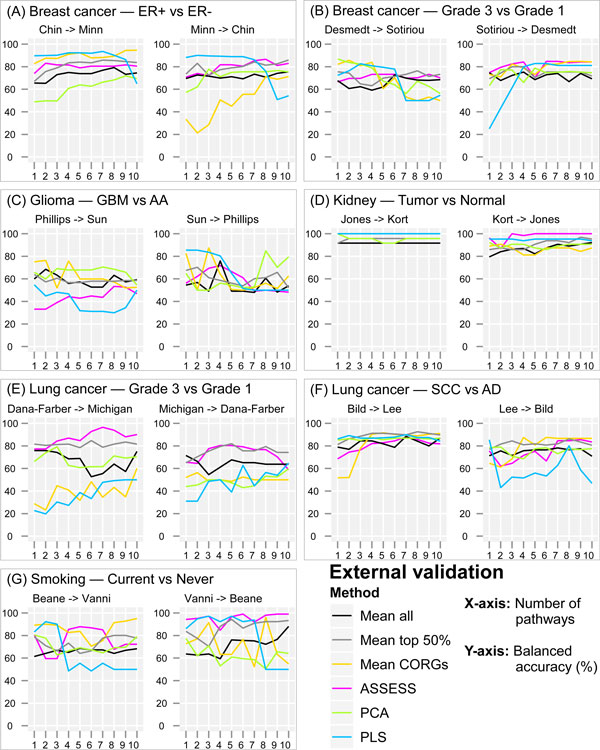
**External validation balanced accuracy in the seven pairs of datasets**. The general format of the plots is the same as in Figure 2. The arrow (- >) in the plot title designates the training and test datasets. For example, "Chin - > Minn" denotes that the classifier was trained on the Chin dataset and tested on the Minn dataset.

Having examined the classification accuracies by cross-validation on the same datasets from which the classifiers were derived, the accuracies were then re-assessed by externally validating on independent datasets with identical phenotypic class labels. As in the internal validation, there were noticeable fluctuations in accuracies along the feature set sizes, and their extent appeared to vary across datasets. For example, the smoking datasets showed the largest fluctuations (Figure 3G), the kidney datasets showed the smallest fluctuations (Figure 3D), and the lung cancer subtype datasets showed medium fluctuations (Figure 3F). As in the internal validation, it appears that there are more pronounced fluctuations in some of the methods (*Mean CORGs*, PCA, and PLS) than in the rest (*Mean all, Mean top 50%*, and ASSESS). Apart from the aforementioned qualitative observations, the presence of the dataset-dependent and feature set size-dependent fluctuations in accuracies makes it difficult to further compare quantitatively the relative performance of the six methods. For example, while it should be relatively straightforward to compare the methods in the breast cancer ER subtype dataset which showed relatively small fluctuations (Figure 3A), it would be difficult to do so in the smoking dataset which showed a high extent of fluctuation (Figure 3G). To facilitate further comparison, a summarized view of accuracies across all the datasets was thus obtained using a rank-based method, as described in the next section.

### Overall accuracy ranking combined across the datasets

**Figure 4:**
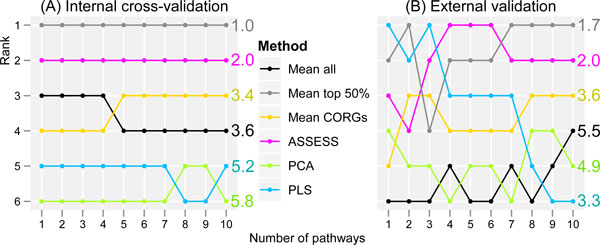
**Overall accuracy ranking of the methods combined across the datasets**. Combined accuracy ranking of each method is plotted as a function of the number of top pathways. The numbers in the right of the plots are the average rank across the ten feature set sizes. (A) Internal cross-validation. (B) External validation.

To simplify the comparison of the methods, it would be helpful to summarize the accuracy results by converting them into a ranking of the six methods in each dataset and combining the rankings from all the fourteen datasets. To this end, we used RankAggreg package [32] which produces a combined ranked list when given with several ranked lists. The rank combination was done for the external validation results as well as for the internal validation results. The combined ranking of the six methods at each feature set size is shown in Figure 4.

With such ranking-based summarized representation, relative performance of the six methods becomes more evident. In the internal validation (Figure 4A), *Mean top 50% *achieved the highest accuracy ranks across all feature set sizes, whereas PCA achieved the lowest accuracy ranks. Overall, the combined accuracy ranking showed only a small extent of fluctuation in the internal validation, despite the fluctuations in accuracy values within individual datasets. In the external validation (Figure 4B), there were considerable fluctuations in the combined accuracy ranking. Nevertheless, relative performance of the six methods was distinguishable despite the fluctuations. By comparing Figure 4B with Figure 4A, it can be observed that the external and the internal validation results are generally similar. First, on average, *Mean top 50% *achieved the highest accuracy rank in both the external validation (average rank of 1.7) and the internal validation (average rank of 1.0). Second, PCA ranked the second lowest in the external validation (average rank of 4.9) and the lowest in the internal validation (average rank of 5.8). Third, *Mean CORGs *achieved a medium accuracy rank in both validations. Fourth, ASSESS achieved the second highest accuracy rank in both validations. On the other hands, disparate observations can also be made between the external and the internal validation results. For example, although PLS ranked consistently near the bottom in the internal validation, it showed a steady decrease in accuracy rank in the external validation as more pathways are added to the classifiers, yielding a medium average rank of 3.3. While the results from the internal and external validation showed both similarities as well as discrepancies, the results from the external validation should receive more weight since the external validation is a more realistic way to estimate the true performance and generalizability of a classifier, as argued in the Introduction section. Therefore, in subsequent sections, further examinations of the classification performance were made to the externally validated results only.

### Overall distribution of accuracies pooled across the datasets and feature set sizes

**Figure 5:**
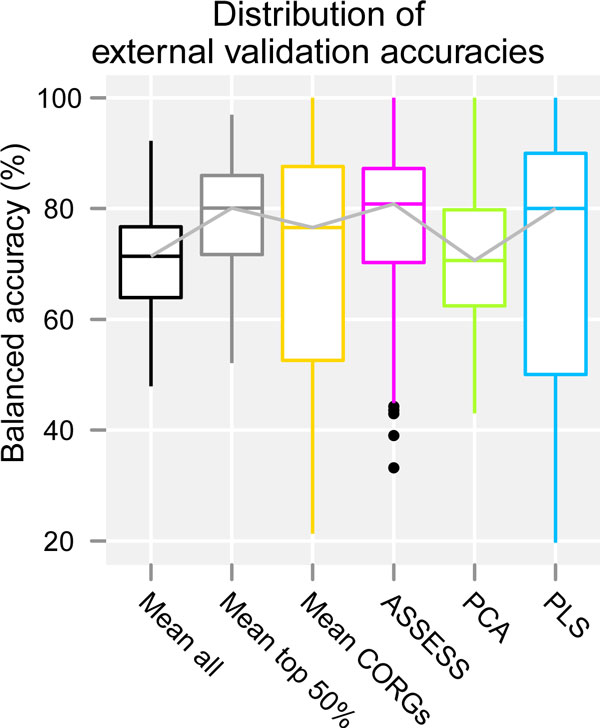
**Overall distribution of accuracies across all the datasets and all feature set sizes**. The distribution was obtained from the external validation results. Median accuracies are joined by grey line.

To further summarize the external validation results shown in Figure 3, overall distribution of accuracies was obtained for each of the six methods by pooling their accuracies obtained under all the fourteen datasets and ten feature set sizes, and represented as boxplots (Figure 5). With respect to median accuracy, three methods (*Mean top 50%*, ASSESS, and PLS) similarly showed a higher accuracy (around 80%), whereas *Mean all *and PCA showed a lower accuracy (around 70%). With respect to dispersion, four methods (*Mean all, Mean top 50%*, ASSESS, and PCA) showed a similar extent of dispersion, whereas *Mean CORGs *and PLS showed a high extent of dispersion, with a small lower quartile value (around 50%). Considering both the median accuracy and the dispersion, *Mean top 50% *and ASSESS thus showed a similarly good performance across all the datasets and feature set sizes. Although PLS showed a good median accuracy, its accuracies varied the most across the datasets and feature set sizes.

### Overall distribution of accuracies in each phenotype

**Figure 6:**
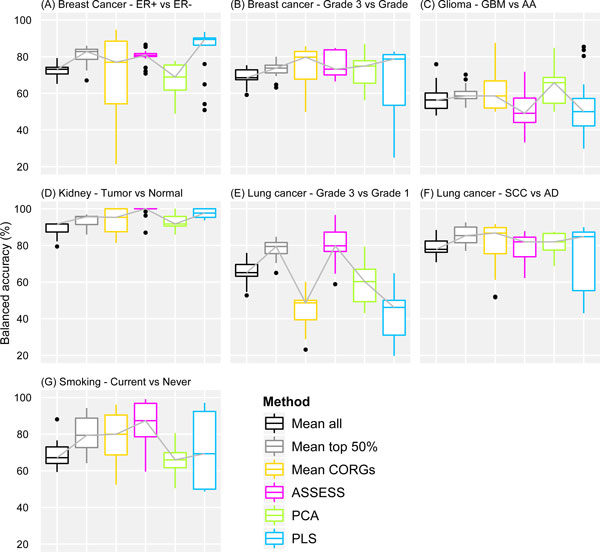
**Overall distribution of accuracies across all feature set sizes in each phenotype**. The format of the plots is the same as in Figure 5. Unlike Figure 5, accuracies were pooled only across the ten feature set sizes and two independent datasets that belong to a phenotype.

The external validation results shown in Figure 3 were also summarized for each of the six methods by pooling their accuracies at all feature sizes and two independent datasets in each phenotype (Figure 6). A main observation is that the relative performance of the methods varies across phenotypes. While the median accuracies of all the methods are relatively similar in some phenotypes (Figures 6B, D, and 6F), there are large differences in other phenotypes (Figures 6E and 6G). What this observation entails is that the performance evaluation should be performed in as many phenotypes as possible, as done in this study.

### Between-dataset correlation of differential expression statistics of pathways

**Figure 7:**
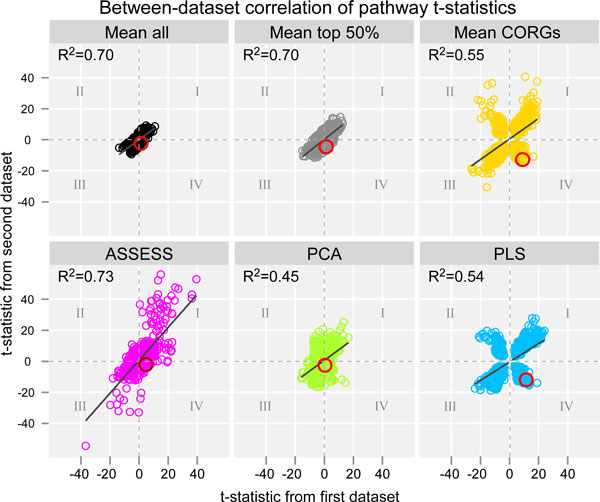
**Between-dataset correlation of pathway t-statistics**. In each scatterplot, Pearson correlation coefficient and linear regression line are shown. Quadrants are indicated as Roman numerals. Pathways in the quadrants II and IV are the discordant pathways. A large red circle denotes an exemplar pathway (MAPK signalling pathway) which is further discussed in the main text. For ASSESS, four outlier data points (with absolute t-statistics of 40 - 70) were removed to keep all six plots under a comparable scale.

Comparison of the methods can also be performed by evaluating the extent to which the differential expression statistics of pathways are correlated between independent datasets. To this end, Student's *t*-test was performed on pathway expression profiles to obtain *t*-statistics of each pathway in each of the datasets. In total, seven pairs of pathway signatures were obtained per method. Thus, for a given phenotype, one can correlate a pathway's *t*-statistic in one dataset with its *t*-statistic in the other dataset. To obtain a summarized view, a scatterplot of *t*-statistics was prepared for each method by correlating *t*-statistic values of all the pathways between seven pairs of datasets (Figure 7). Thus each plot contains 1,141 data points (163 pathways times 7 phenotypes). Two main observations can be made from the scatterplots. First, lower correlations were observed under three methods (*Mean CORGs*, PCA, and PLS) than the rest (*Mean all, Mean top 50%*, and ASSESS). Second, the three methods with lower correlations have many discordant pathways whose *t*-statistics are large in magnitude and in the opposite sign between the two independent datasets (quadrants II and IV). It is these discordant pathways that, when used as a feature, dropped the external validation accuracy and caused the observed fluctuation of accuracies shown in Figure 3.

**Figure 8:**
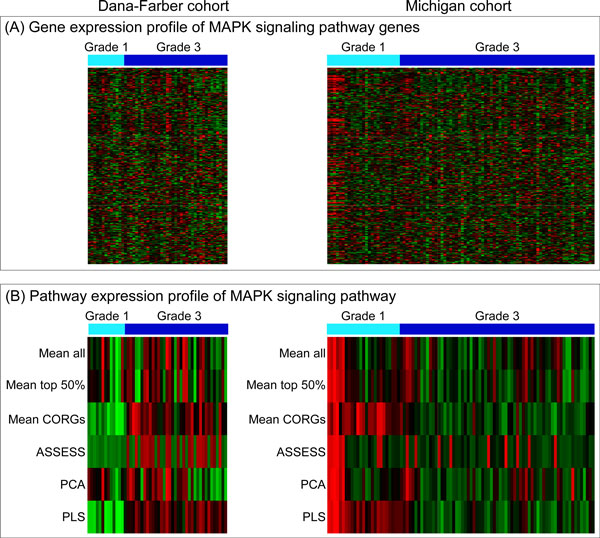
**Expression profiles of MAPK signalling pathway as an example of discordant pathway**. Expression profiles of MAPK signalling pathway in the two cohort datasets of lung cancer grade are shown. (A) Expression profile of 244 member genes. (B) Pathway expression profiles aggregated by the six methods. In grade 3, the pathway shows an up-regulation in the Dana-Farber cohort but a down-regulation in the Michigan cohort.

To further examine the discordant pathways, an exemplar pathway was selected among the top 10 feature pathways used in the classification analysis. Since the lung cancer grade datasets showed lower accuracies (Figure 6E) under the three methods (*Mean CORGs*, PCA, and PLS) than the rest, an exemplar pathway was selected from the feature pathways in the lung cancer grade datasets. One of the most discordant pathways with the largest magnitude of *t*-statistic values was MAPK signalling pathway, whose 244 member genes are represented in the microarray data. In *Mean CORGs*, it was the most up-regulated pathway in one dataset (Dana-Farber) but fifth most down-regulated pathway in the other (Michigan) (data not shown). In PLS, it was also the most up-regulated pathway in the Dana-Farber dataset but ranked the twenty-first as a down-regulated pathway in the Michigan dataset (data not shown). Its *t*-statistics in the lung cancer grade datasets are indicated as large red circles in Figure 7, and its member genes' expression profile along with its pathway expression profiles aggregated by the six methods are shown in Figure 8. Three observations can be made from Figure 8. First, differential expression between the grade groups becomes more evident with pathway expression profiles (Figure 8B) than gene expression profile (Figure 8A). Second, the direction of pathway-level expression change is discordant between the two cohort datasets. In the grade 3 samples, the MAPK signalling pathway is up-regulated in the Dana-Farber cohort (Figure 8B, left) but down-regulated in the Michigan cohort (Figure 8B, right). Third, the extent of differential expression of the pathway is more pronounced under *Mean CORGs *and PLS, as all the samples within each grade group show relatively homogeneous expression levels.

## Discussion

This study evaluated and compared the six methods of pathway-level aggregation of gene expression data--five existing methods (*Mean all, Mean CORGs*, ASSESS, PCA, and PLS) and a simple variant of an existing method (*Mean top 50%*). Four of the compared methods are either conventional dimensionality reduction methods (PCA and PLS) or simple heuristics (*Mean all *and *Mean top 50%*). These four methods were not specifically developed to address the pathway-level aggregation problem itself. It may be argued that the former lacks a solid biological basis and the latter a formal basis. The other two methods (*Mean CORGs *and ASSESS) were specific to the pathway-level aggregation problem. The evaluation was performed with respect to classification accuracy, validated both internally and externally, and by examining the correlative extent of pathway signatures between seven pairs of related but independent datasets. The results show that there are considerable differences in performance between the methods. Three main findings from this study are described below. First, with respect to the external validation accuracy, the six methods can be ranked from the best to the worst as *Mean top 50%*, ASSESS, PLS, *Mean CORGs*, PCA, and *Mean all *(Figure 4B). The dimensionality reduction methods were overall inferior to others. Second, the overall distribution of accuracies is skewed down in *Mean CORGs *and PLS (Figure 5), indicating that there are many instances in which these two methods did not perform well. Third, the reason why these two methods perform poorly in such instances is that they tend to produce pathway expression signatures that are discordant between related but independent datasets, as shown in the pathway *t*-statistic correlation (Figure 7). The between-dataset discordance arises due to the algorithmic characteristics of the two methods. In *Mean CORGs*, the pathway's overall direction of expression change is first determined as the sign of mean *t*-statistic values of all its member genes. Then, only the key member genes that contribute the most to the pathway-level *t*-statistic are selected. Thus, if the pathway's overall expression change is in different directions between datasets even by a small magnitude, the discordance in the resultant pathway expression profiles becomes inflated. Similarly, in PLS, the sign of the latent components was corrected so that the component scores are positively correlated with the average gene expression profile of the given pathway. Thus, the pathway expression profile aggregated by PLS becomes discordant in the opposite direction as well.

The third observation described above suggests that, in order to yield a pathway expression profile that is concordant between independent datasets and predictive of a phenotype as well, a successful pathway-level aggregation method should not strictly depend on the overall direction of expression change of a pathway, as measured by the mean of *z*-scaled expression levels of its member genes. Although it is the simplest and the most widely used practice that regards a pathway being up-regulated if the mean expression is positive and down-regulated if negative, such a rationale lacks solid biological support. It has been argued that, in physiological processes such as biological pathways, homeostatic transcriptional responses may occur so that an up-regulation of one component in a pathway leads to a down-regulation of another component in that pathway in an attempt to compensate [33]. If so, overall direction of a pathway's expression change is determined by the relative magnitudes of the two opposing components. If, for some reason, the relative magnitudes of the two components are reversed between independent datasets, the overall direction of a pathway's expression change would become discordant in the opposite direction. Furthermore, simply averaging the expression changes of all the member genes as if they were independent and uniform objects may not lead to an adequate measure for representing a pathway's expression behavior in a biological context, considering that differential expression of downstream genes in a pathway may not influence the pathway's overall activity as much as that of its upstream genes [34]. Thus, I speculate that a successful pathway-level aggregation method needs to consider the issues such as the homeostatic response of a pathway, the positions of genes in the pathway topology, as well as the types of interaction linking the member genes. The last two issues have also been mentioned in a review paper describing topology-based pathway enrichment analysis methods [35].

The pathway-level aggregation approach is advantageous in that it can be used not only for identifying differentially expressed pathways, but also for classification or clustering samples in the space of pathways, for deriving a compact representation and visualization of the original gene expression data, as well as for facilitating systems-level interpretation. Nevertheless, an inherent problem of the pathway-level aggregation approach is a possible loss or even deformation of information present in the original gene expression data. This problem arises since the expression levels of pathway member genes are reduced into a single numerical value. In this regard, a relationship may exist between the discriminative ability of a pathway expression profile and the size of the pathway, since the size of a pathway may indicate either complexity or extent of characterization of the pathway. To examine whether such a relationship exists, for each of the fourteen datasets, the 163 pathways were ranked by Student's *t*-test *p*-value. At each rank, a box plot was prepared to summarize the sizes of the pathways occupying that rank in the fourteen datasets. Then the median pathway sizes are joined to show the overall trend between size and rank (Additional file 2). Overall, there were no strongly discernible trends that are suggestive of the size-rank relationship of pathways. Another potential problem of the pathway-level aggregation approach is a possible correlation between pathways whose member genes partially overlap. For such pathway pairs, a pathway that is actually irrelevant to the given phenotype may assume a discriminative expression profile merely because it shares many member genes with the other relevant and genuinely discriminative pathway. It is desirable to avoid identifying such an irrelevant pathway as discriminative since it would otherwise confound the interpretation of the results. In the domain of functional enrichment analysis, this problem has been addressed by approaches such as performing separate analyses on the shared and unique member subsets in the pathways [36], and giving smaller weights to the genes that assume intermediate positions in the pathway network topology, which are likely to be shared among pathways [37]. Similar lines of approach may be possible in the domain of pathway-level aggregation.

There are four major strengths in the evaluation scheme adopted in this study, which raise validity of the reported results. First, the evaluation is comprehensive due to the use of seven pairs of related but independent datasets. Evaluating the performance under a multitude of datasets encompassing various phenotypes helps to draw a generalized conclusion. As shown in Figure 6, both the median and the distribution of accuracies varied across the seven phenotypes. If only the datasets from a couple of phenotypes had been used for benchmarking (for example, Figures 6D and 6F), it would have been incorrectly concluded that the six compared methods did not differ in their median accuracy. Second, the evaluation is stringent by externally validating classification accuracy on independent datasets. Third, the evaluation is transparent since the classification accuracy was examined as a function of feature set size (from top one to ten pathways, ranked by *t*-test on pathway-level aggregated data), instead of at a fixed arbitrary number of features, or an optimized number of features that results in the best performance in the validation dataset [8]. While the resultant fluctuations in accuracy across the feature set sizes (Figure 3) may make the performance comparison less evident, they transparently show that the expression profile of top-ranked differentially regulated pathways might be discordant between datasets. Fourth, the evaluation was also done with respect to the reproducibility of pathway signatures by examining correlation of pathway *t*-statistics between datasets. Its results further substantiate the discordance of pathway signatures between datasets, especially under *Mean CORGs *and PLS-based aggregation.

There are also three minor contributions in this study. First, the collection of seven pairs of related but independent gene expression datasets can be used for benchmarking analysis of any methods based on gene expression data. Second, the sign ambiguity of PCs in PCA has been explicitly stated and resolved. Some of the previous studies that used PCA as an aggregation method either overlooked the sign ambiguity issue [6,7], or simply pointed out that the PCA-aggregated expression profile is not readily interpretable since it does not capture the direction of expression changes of pathways [4]. The present study explicitly stated the issue and followed a simple available approach that corrects the signs of PC scores so that the PC scores are positively correlated with the pathway's average gene expression profile [15,17]. An alternative solution [16] has also been proposed in the field of chemometrics, in which the sign of PCs are determined by computing the sign of the sum of inner products between PC and all data points in the dataset. A preliminary analysis employing this approach did not result in better classification accuracy (data not shown), thus it was not considered further in the analysis. Third, the present study also explicitly stated and resolved the sign correction issue of latent components in PLS, which has been neither stated nor resolved in a previous study that used PLS as a pathway-level aggregation method [4].

In light of the findings obtained from this study, it appears that there is both room and necessity for developing a novel method for pathway-level aggregation. On one hand, considering that averaging the expression profiles of all pathway member genes (that is, *Mean all*) results in the lowest accuracy, it is remarkable that simply removing half of the genes with lower *t*-statistics (that is, *Mean top 50%*) leads to the highest accuracy (Figure 4B). On the other hand, any method that is dependent on the pathway's overall change direction, as in *Mean CORGs *and PLS, is likely to manifest the discordance between independent datasets. This issue needs to be considered and resolved in developing a novel method, since one of the expectations in pathway-level aggregation has been that the expression of the pathway as a whole may become more consistent, predictive, and reproducible across independent datasets despite gene-level inconsistencies [5,9].

## Conclusions

In conclusion, this study evaluated and compared the six methods for pathway-level aggregation of gene expression data. Comprehensive and stringent evaluation was made possible by collecting seven pairs of related but independent datasets encompassing various phenotypes. The evaluation was performed with respect to classification accuracy, validated both internally and externally, and by examining the correlative extent of pathway signatures between the dataset pairs. The best accuracy and correlation were obtained when pathway-level expression profiles were derived by ASSESS and *Mean top 50%*. Thus these two methods are suggested to be the preferable solutions. The lowest accuracy was obtained from *Mean all*. PLS and *Mean CORGs *gave rise to the largest extent of discordance in the pathway signature correlation. The analysis shown in this report also implies that there is both room and necessity for developing a novel method for pathway-level aggregation.

## Methods

### Collection of benchmarking datasets

The seven pairs of independent datasets were collected by searching the public repositories and the literature under the following criteria. First, the search was confined to human datasets to simplify the data processing and analysis. Second, only the two-class unpaired datasets were considered for inclusion into the collection. Third, only the datasets employing relatively recent Affymetrix platforms were considered, that is, excluding cDNA platforms or older Affymetrix platforms such as Human Genome U95. The reason was to ensure a good coverage of genome and thus a reliable representation of a pathway's member set. Fourth, any of the two classes was required to contain ten or more samples to facilitate internal cross-validation. Fifth, and most importantly, there should be two related but independent datasets with identical phenotypic class labels under investigation (for example, two datasets that compared lung tissue between smokers and non-smokers), generated from independent laboratories.

### Pre-processing of gene expression data

Data were downloaded from public repositories indicated in Table 1. The data were obtained as CEL files in all datasets, except for the two datasets on lung cancer grade in which the data are available only as Gene Expression Omnibus (GEO) series matrix files. The CEL files were pre-processed by RMA method and log_2_-transformed. The GEO series matrix data were already in an RMA-processed form (GSE3141) or a GCRMA-processed form (GSE8894). Expression data from probesets without Entrez and gene symbol annotations (based on Affymetrix annotation release 30) were discarded. Then expression levels from multiple probesets representing the same gene were averaged, yielding gene-level expression profile data containing the following numbers of unique genes: 13,029 genes (U133A and U133A 2.0); 20,315 genes (U133 Plus 2); and 18,485 genes (U133 Set). For three of the seven phenotypes, a pair of independent experiments was performed in different platforms. In such cases, the genes that are commonly represented in both platforms were selected, which were a subset of one of the platforms with larger coverage (that is, genes in U133A were a subset of the genes in U133 Plus 2). Then only the expression data of the common genes were used for the analysis of the corresponding phenotype.

### Pathway-level aggregation of gene expression data

The gene expression data were aggregated at KEGG pathway level. The list of KEGG pathways and their member genes was obtained from MSigDB version 3.0 [38] which contained a total of 186 KEGG pathways. Among them, there were 163 pathways consisting of at least 20 and at most 300 member genes, which were used for subsequent pathway-level aggregation. For the three mean-based aggregation methods (*Mean all, Mean top 50%*, and *Mean CORGs*), an R code was written to implement them. For ASSESS, its java program was used under its default parameter setting. For PCA and PLS, *moduleEigenegenes *function in WGCNA package [39] and pls package [40] were used, respectively. For both PCA and PLS, the signs of elements in the component score vector were corrected so that the score vector is positively correlated with average gene expression profile of the given pathway. Prior to aggregation, gene expression data were *z*-scaled for all the methods, except for ASSESS which does not require its input to be *z*-scaled.

### Selection of features used for classification

In each of the fourteen datasets, features (that is, pathways) were first ranked by their *p*-values from Student's *t*-test on the pathway-level expression data. Top pathways were then selected, whose expression values were used for subsequent training of classifiers and their performance evaluation. The number of selected pathways was varied from one to ten in order to transparently assess the classifier's performance across a range of features.

### Consideration of class imbalance

All the fourteen datasets showed class imbalance--there were more samples in case group than in control group. With such datasets, a majority-class classifier is often obtained since conventional misclassification error rate can be made meaninglessly low simply by predicting all samples as a case sample. To correct for the class imbalance problem, three measures were taken--two in classifier training and one in performance evaluation, as described in the next two sections.

### Parameter tuning of SVM classifier

Classification was performed with SVM implemented in the e1071 package. Radial basis function was chosen as a kernel. There are two SVM parameters that affect decision boundary and thus need to be tuned. They are cost and gamma whose default values in the e1071 package are 1 and 1/number of selected features, respectively. In the current analysis, the parameter tuning was performed by a grid search in which the cost parameter was varied as C = 2^-4^, 2^-3^, ..., 2^6 ^and the gamma range was the cost values divided by the number of selected features. To perform grid search, 5-fold cross-validation was performed to select the best pair of the parameter values that minimizes an error function, which was subsequently used to train classifiers for validation of classification performance. During the grid search, two measures were taken in order to guard against the class imbalance problem. First, class weight parameter (*class.weights*) was set to the inverse of class proportion, instead of the default equal weights, in order to compensate for the effect of unbalanced class proportions. Second, error function parameter (*error.fun*) was set to average classification error, which is the mean of false positive rate and false negative rate, instead of the default misclassification rate, which is an overall proportion of all the misclassified samples. Since the average classification error is the mean of the two error rates, a majority-classifier that classifies most of the samples as positives would get a high false positive rate and thus a high average classification error.

### Balanced accuracy as the measure of classification performance

As in the training phase, a measure was taken to guard against the class imbalance problem by adopting balanced accuracy as the performance measure. The balanced accuracy is defined as the mean of true positive rate and true negative rate. As such, a majority-classifier would get a low true negative rate and thus a low balanced accuracy, whereas its conventional accuracy (overall proportion of correct classifications) can be meaninglessly high.

### Validation of classification performance

Using the balanced accuracy as the performance measure, a stringent evaluation of classification performance was performed, which consists of both internal and external validations. For the internal validation, 5-fold cross-validation was performed in a stratified manner such that class proportions in each training set are kept the same as in the original dataset. At each iteration of cross-validation, pathway-level aggregation and feature selection were performed anew, as has been emphasized as being the correct practice of cross-validation [41,42]. To achieve more stable cross-validation results, any possible effect of a particular random split needs to be avoided. Thus the cross-validation was repeated twice to obtain a mean balanced accuracy averaged over five folds and two repetitions. Only two repetitions were achievable since the ASSESS program is available as a GUI implementation instead of a command line version and since a single iteration of the cross-validation experiment alone involves a total of seventy training-test set pairs (five folds times fourteen datasets) each of which need to be aggregated at pathway-level by the ASSESS and all other methods. For the external validation, a classifier was trained on one dataset and tested on the other dataset for each of the seven pairs of independent datasets.

### Summarization of performance rankings across the datasets

**Figure 9:**
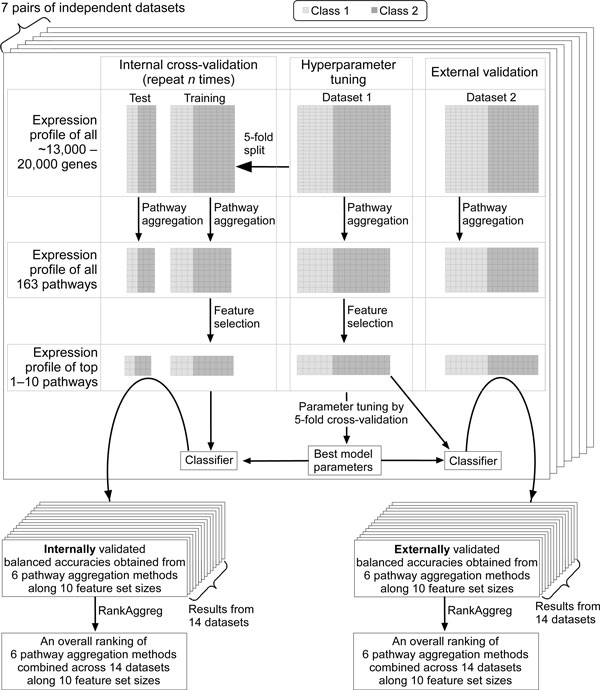
**Illustration of analysis steps**. Hyperparameter tuning, internal and external validations, and rank aggregation of the validation results are depicted.

For each of the fourteen datasets, six methods were ranked according to their balanced accuracy values. The resultant fourteen ranked lists of six methods were combined into a single ranked list with RankAggreg package [32]. In brief, accuracies from the six methods were ranked from the highest to the lowest in each of the fourteen datasets. At each feature set size, a total of fourteen ranked lists were thus obtained. Each of the lists arranges the six methods according to their accuracies at the given feature set size and dataset. At each feature set size, a combined ranking of the methods was then obtained by merging the fourteen ranked lists with the RankAggreg package. Using the package, brute force rank summarization was performed with balanced accuracies as weights and Spearman footrule distance as a distance measure. All the aforementioned analysis steps are depicted in Figure 9.

### Softwares

In addition to the aforementioned softwares for pathway-level aggregation, classification, and rank aggregation, the analysis performed in this paper used R and Perl for numerical computation and data parsing. All plots were generated using ggplot2 R package.

## Competing interests

The author declares that they have no competing interests.

## Authors' contributions

SH conceived of the project, analyzed the data, and wrote the manuscript.

## Supplementary Material

Additional File 1 **Supplementary Methods**. This document contains the schematic and mathematical description of the pathway-level aggregation methods. Click here for file

Additional File 2 **Relationship between size and rank of pathways**. The 163 pathways, whose sizes are between 20 and 300, are ranked by Student's *t*-test *p*-value in all fourteen datasets. Smaller ranks correspond to more significance. For each rank, a box plot was prepared to summarize the sizes of the pathways at that rank in the fourteen datasets. Median sizes are joined by thick line to show the overall size-rank trend. Click here for file
