# Supplementary material for: Comparison and evaluation of pathway-level aggregation methods of gene expression data
Source: BMC Genomics. 2012 Dec 7;13(Suppl 7):S26. doi: 10.1186/1471-2164-13-S7-S26 (PMC3521227; doi:10.1186/1471-2164-13-S7-S26)
Supplement: Additional File 1 — Supplementary Methods. This document contains the schematic and mathematical description of the pathway-level aggregation methods. [file 1471-2164-13-S7-S26-S1.pdf]

Additional file 1: Schematic and mathematical description of the pathway-level aggregation methods

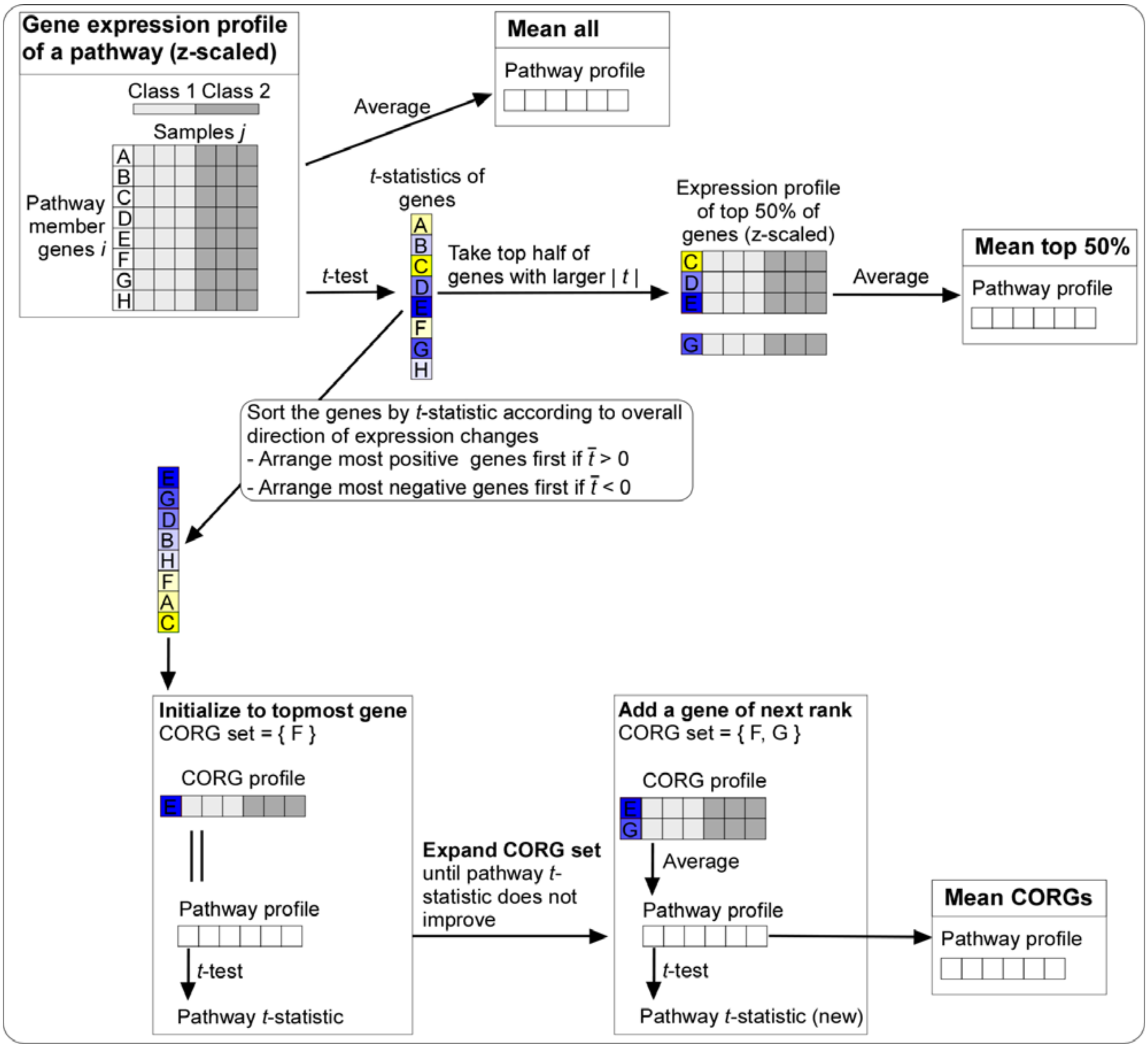

Schematic of the three mean-based methods. Algorithmic steps in *Mean all*, *Mean top 50%*, and *Mean CORGs* are schematized.

Mathematical description of the mean-based methods

Given a gene expression data with n samples and a pathway whose m member genes are represented in the data, let an m x n matrix **X** be a z-scaled gene expression profile of the pathway's member genes. Then, each element x_{ij} is a z-scaled expression level of a member gene i in sample j. Pathway-level aggregation methods seek to derive a pathway expression profile **a** which is a vector with n elements.

Mean all

Each element a_j is calculated as

$$a_j = \frac{1}{m}\sum_{i=1}^{m} x_{ij} \tag{1}$$

Mean top 50%

The member genes' expression profile is subject to Student's t-test. Then, the member genes are sorted by $|t|$ in descending order, or equivalently, by p-value in ascending order. The top 50% of the member genes are selected, and their gene expression profile is averaged as in Equation (1).

Mean CORGs

The member genes' expression profile is subject to Student's t-test. Overall direction of the pathway's expression change is found by the sign of the mean of all the member genes' t-statistics ($\bar{t}$). Then, the member genes are sorted by t-statistic according to the overall direction;

Descending order if $\bar{t} > 0$ (Most up-regulated genes are arranged to the top)

Ascending order if $\bar{t} < 0$ (Most down-regulated genes are arranged to the top)

In this way, a sorted list of member genes {g1, g2, g3, …, gm} is obtained.

Let G_k be a set of CORGs containing top k member genes. Then each element a_j is given by;

$$a_j = \frac{1}{\sqrt{k}}\sum_{i=1}^{k} x_{ij} \tag{2}$$

where the sum is divided by square root of k to stabilize variance.

Let $S(G_k)$ the pathway-level t-statistic obtained from **a**. Finding CORG set amounts to identify optimal k member genes that maximize the pathway-level t-statistic.

The CORG set is iteratively expanded until the pathway-level t-statistic does not improve, at which point the final CORG set and its aggregated pathway expression profile **a** is returned, as shown in the pseudocode;

```
Initialize G0 = { } and S(G0) = 0
FOR i = 1 to m
    Add the next ranked gene gi to CORG set Gi
    Aggregate the member genes' expression by Equation (2) to obtain a
    Perform t-test on a to obtain S(Gi)
    IF |S(Gi)| < |S(Gi-1)|
        BREAK
END FOR
```

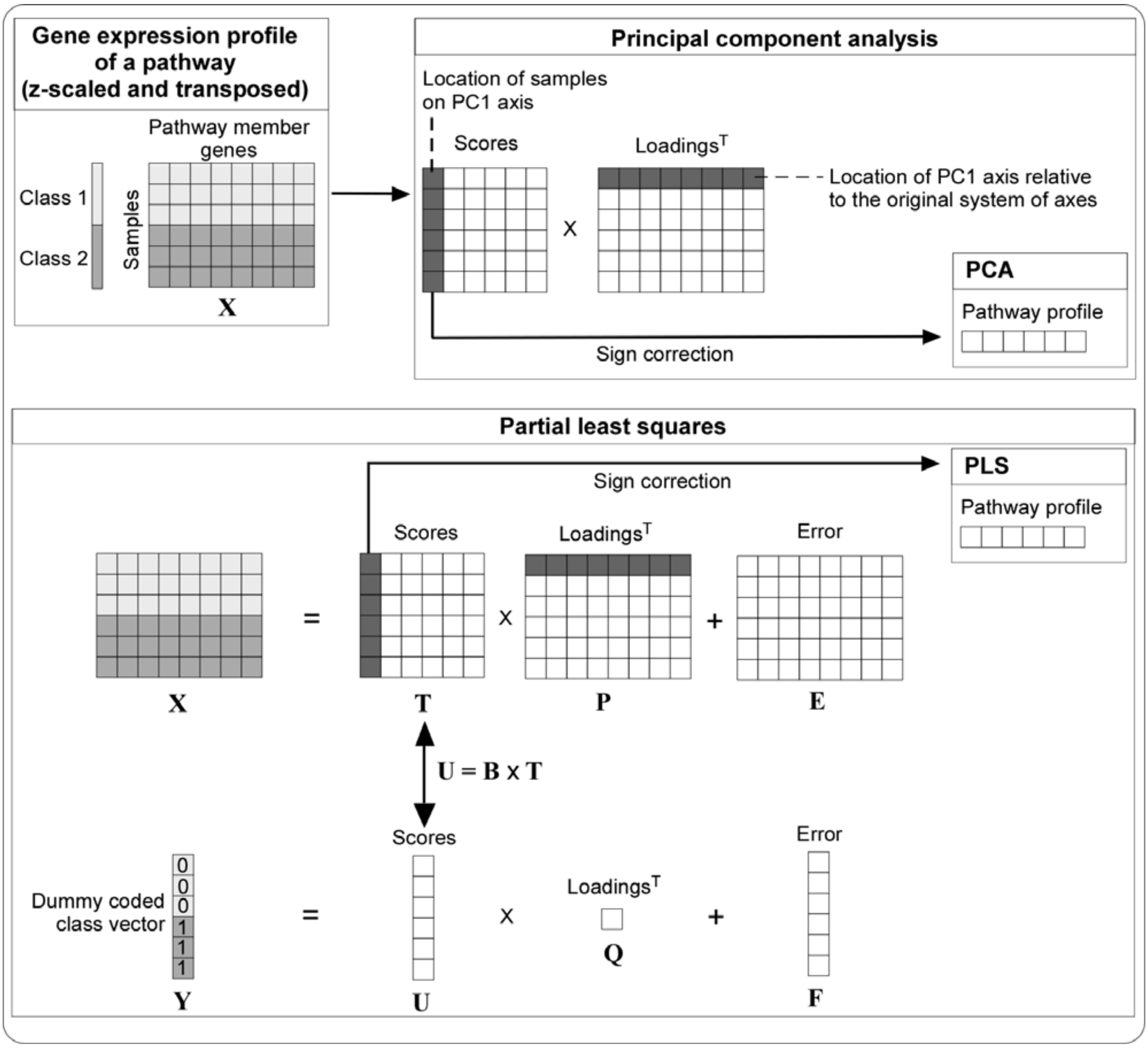

Schematic of the two projection-based methods. Algorithmic steps in PCA and PLS are schematized.

Mathematical description of the projection-based methods

PCA (Principal Component Analysis)

PCA expects a data matrix in which samples are arranged in rows and variables in columns. Thus the aforementioned m x n matrix **X** needs to be transposed to an n x m matrix so that samples are arranged in rows and genes in columns. To simplify notation, the transposed matrix $\mathbf{X}^{\mathrm{T}}$ will be referred to simply as **X** from now on.

Method 1. PCA by singular value decomposition (SVD) of X

PCA can be performed by SVD of **X**, which yields the factorization

$$\mathbf{X} = \mathbf{U\Sigma V}^{\mathrm{T}} \tag{3}$$

where

U is an n x n orthogonal matrix

Σ is an n x n diagonal matrix

V is an m x n orthogonal matrix.

The matrix product **UΣ** is called the scores, in which each column gives the location of n samples with each PC axis. The matrix **V** is called the loadings, in which each column gives the location of each PC axis relative to the original system of m axes. First column in the scores matrix is taken as the pathway expression profile vector **p**.

Method 2. PCA by eigenvalue decomposition of a covariance matrix of X

Alternatively, PCA can be performed by eigenvalue decomposition of a covariance matrix of **X**.

An m x m symmetric matrix **C** which is given by the following equation

$$\mathbf{C} = \frac{1}{n-1}\mathbf{X}^{\mathrm{T}}\mathbf{X} \tag{4}$$

is called the covariance matrix of **X** (if **X** is mean-centered) or correlation matrix of **X** (if **X** is mean-centered and divided by standard deviation; i.e., z-scaled).

Since **C** is a symmetric matrix, **C** is an orthogonal matrix and orthogonally diagonalizable. Thus, **C** has n linearly independent eigenvectors **p** such that

$$\mathbf{Cp}_i = d_i\mathbf{p}_i, \qquad i = 1,\ldots,m \tag{5}$$

where $\mathbf{p}_{\mathrm{i}}$ is i-th eigenvector and d_{i} is corresponding eigenvalue.

In matrix form, Equation (5) can be written as

$$\mathbf{CP} = \mathbf{PD} \tag{6}$$

where $\mathbf{D} = \mathrm{diag}\{d_1,\ldots,d_m\}$

Since **P** is an orthogonal matrix, it holds that $\mathbf{P}^{\mathrm{T}} = \mathbf{P}^{-1}$. Thus Equation (6) can be written as

$$\mathbf{C} = \mathbf{PDP}^{\mathrm{T}} \tag{7}$$

where

P is an m x m orthogonal matrix whose columns are eigenvectors of **C**

D is an m x m diagonal matrix whose diagonal entries are eigenvalues of **C**.

Relationship between the two methods

It can be seen that the two aforementioned approaches yield the same results as shown below.

From Equation (3), $\mathbf{X}^\mathrm{T}\mathbf{X}$ is given by

$$\mathbf{X}^\mathrm{T}\mathbf{X} = \left(\mathbf{U\Sigma V}^\mathrm{T}\right)^\mathrm{T}\left(\mathbf{U\Sigma V}^\mathrm{T}\right) = \left(\mathbf{V\Sigma U}^\mathrm{T}\right)\left(\mathbf{U\Sigma V}^\mathrm{T}\right) = \left(\mathbf{V\Sigma}\right)\left(\mathbf{U}^\mathrm{T}\mathbf{U}\right)\left(\mathbf{\Sigma V}^\mathrm{T}\right) = \left(\mathbf{V\Sigma}\right)\left(\mathbf{I}\right)\left(\mathbf{\Sigma V}^\mathrm{T}\right) = \mathbf{V\Sigma}^2\mathbf{V}^\mathrm{T}$$

From Equations (4) and (7), $\mathbf{X}^\mathrm{T}\mathbf{X}$ is given by

$$\mathbf{X}^\mathrm{T}\mathbf{X} = \left(n-1\right)\mathbf{C} = \left(n-1\right)\mathbf{PDP}^\mathrm{T}$$

Thus, it follows that $\mathbf{V} = \mathbf{P}$ and $\left(n-1\right)\mathbf{D} = \mathbf{\Sigma}^2$.

How to perform PCA in R

For the *z*-scaled and transposed *n* x *m* matrix **X**, PCA can be performed by either `prcomp()` or `svd()`, yielding the same results. First column of the resultant scores matrix is taken as the pathway expression vector **a**.

Using prcomp()

```
    PCA <- prcomp(X, center=F, scale=F)
    Scores <- PCA$x
    PathwayExpressionVector <- Scores[,1]
```

Using svd()

```
SVD <- svd(X)
    U <- SVD$u
    D <- diag(SVD$d)
    Scores <- U %*% D
    PathwayExpressionVector <- Scores[,1]
```

In the analysis shown in the paper, `moduleEigengenes()` function in WGCNA package was used, which use `svd()`. To correct the sign of the elements in the pathway expression vector **a**, the function was called with the `align` parameter as follows;

```
    dummyColors <- rep("grey", numberOfMemberGenes)
    ME <- moduleEigengenes(X, align="along average", scale=F, color=dummyColors)
    PathwayExpressionVector <- ME$eigengenes[[1]]
```

PLS (Partial Least Squares)

PLS seeks to find a regression model between **T** and **U** (the principal component scores of **X** and those of **Y**, respectively).

The matrix **X** is decomposed into a score matrix **T** and a loading matrix **P**, and an error term **E**. The matrix **Y** is decomposed into a score matrix **U** and a loading matrix **Q**, and an error term **F**. In two-class classification problems, the matrix **Y** is a dummy coded class vector. The goal of PLS is to minimize the norm of **F** while keeping the correlation between **X** and **Y** by the relation **U** = **BT**.

How to perform PLS in R

For the *z*-scaled and transposed *n* x *m* matrix **X**, and a dummy coded class vector **Y**, PLS can be performed by pls package. First column of the resultant scores matrix is taken as the pathway expression vector **a**. Sign correction can be done by using 0(control)/1(case) coding for an overall up-regulated pathway and 1(control)/0(case) coding for an overall down-regulated pathway.

```
Data <- data.frame(Y, X)
PLS <- plsr(Y~X, ncomp=2, data=Data, validation="LOO") #ncomp value does not
matter since we use only the first component
PathwayExpressionVector <- PLS$scores[,1]
```

Mathematical description of the ASSESS method

Since this algorithm is comparably complex, interested readers are advised to refer to the original article for a precise mathematical description of the algorithm (Edelman E, Porrello A, Guinney J, Balakumaran B, Bild A, Febbo PG, Mukherjee S: Analysis of sample set enrichment scores: assaying the enrichment of sets of genes for individual samples in genome-wide expression profiles. *Bioinformatics* 2006, 22:e108-e116)
