# Supplementary material for: Comparison and evaluation of pathway-level aggregation methods of gene expression data
Source: BMC Genomics. 2012 Dec 7;13(Suppl 7):S26. doi: 10.1186/1471-2164-13-S7-S26 (PMC3521227; doi:10.1186/1471-2164-13-S7-S26)

Additional file 2: Relationship between size and rank of pathways. The 163 pathways, whose sizes are between 20 and 300, are ranked by Student's t-test p-value in all fourteen datasets. Smaller ranks correspond to more significance. For each rank, a box plot was prepared to summarize the sizes of the pathways at that rank in the fourteen datasets. Median sizes are joined by thick line to show the overall size-rank trend.

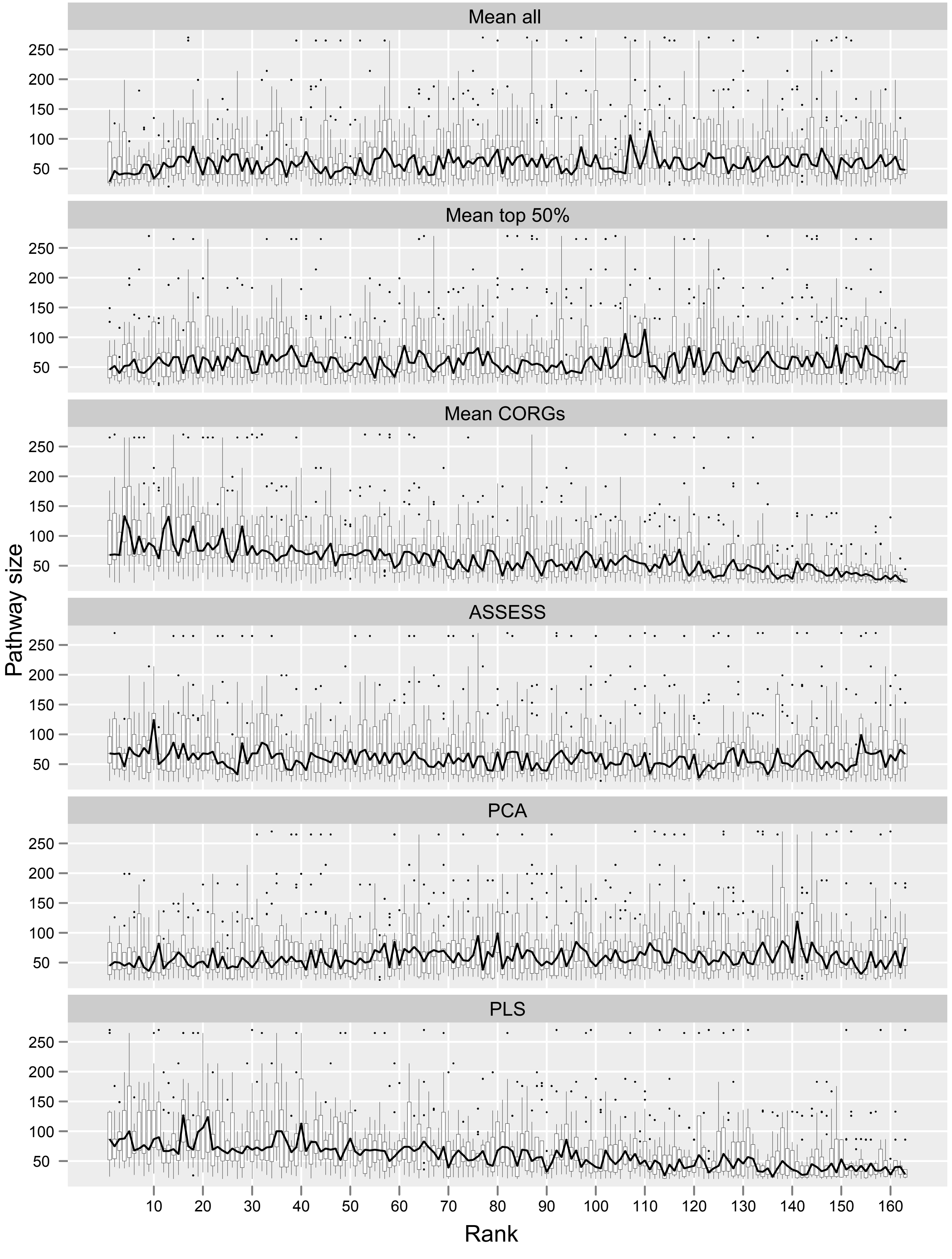

Supplement: Additional File 2 — Relationship between size and rank of pathways. The 163 pathways, whose sizes are between 20 and 300, are ranked by Student's t-test p-value in all fourteen datasets. Smaller ranks correspond to more significance. For each rank, a box plot was prepared to summarize the sizes of the pathways at that rank in the fourteen datasets. Median sizes are joined by thick line to show the overall size-rank trend. [file 1471-2164-13-S7-S26-S2.pdf]
